# Non-sliding Fixation Shows Improved Clinical Outcomes for Displaced Femoral Neck Fractures as Compared to Sliding Fixation

**DOI:** 10.3389/fsurg.2022.826159

**Published:** 2022-03-24

**Authors:** Xiangyu Xu, Yang Lv, Zengzhen Cui, Jixing Fan, Fang Zhou, Yun Tian, Hongquan Ji, Zhishan Zhang, Yan Guo, Zhongwei Yang, Guojin Hou

**Affiliations:** ^1^Department of Orthopedics, Peking University Third Hospital, Beijing, China; ^2^Engineering Research Center of Bone and Joint Precision Medicine, Ministry of Education, Beijing, China

**Keywords:** femoral neck fracture, three cannulated screws, four cannulated screws, surgical method, clinical outcomes

## Abstract

**Objective:**

To compare the clinical outcomes between use of sliding fixation (three cannulated screws, TCS) and non-sliding fixation (four cannulated screws, FCS) in the treatment of femoral neck fractures.

**Methods:**

We retrospectively analyzed 102 patients with fresh femoral neck fractures treated with TCS (60 cases) and FCS (42 cases) between January, 2018 and December, 2019. The demographic data, follow-up time, hospitalization time, operation time, blood loss, length of femoral neck shortening (LFNS), soft tissue irritation of the thigh (STIT), Harris hip score, and complications (such as internal fixation failure, non-union, and avascular necrosis of the femoral head) were also collected, recorded, and compared between the two groups.

**Results:**

A total of 102 patients with an average age of 60.9 (range, 18–86) years were analyzed. The median follow-up time was 25 (22 to 32) months. The LFNS in the FCS group (median 1.2 mm) was significantly lower than that in the TCS group (median 2.8 mm) (*P* < 0.05). In the Garden classification, the number of displaced fractures in the TCS group was significantly lower than that in the FCS group (*P* < 0.05). The median hospitalization time, operation time, blood loss, reduction quality, internal fixation failure rate (IFFR), STIT, and Harris hip score were not statistically different between the two groups (*P* > 0.05). However, in the subgroup analysis of displaced fractures, the LFNS (median 1.2 mm), STIT (2/22, 13.6%), and Harris hip score (median 91.5) of the FCS group at the last follow-up were significantly better than the LFNS (median 5.7 mm), STIT (7/16, 43.8%), and Harris hip score (median 89) of the TCS group (*P* < 0.05). No complications such as incision infection, deep infection, pulmonary embolism, or femoral head necrosis were found in either group.

**Conclusion:**

TCS and FCS are effective for treating femoral neck fractures. For non-displaced fractures, there was no significant difference in the clinical outcomes between the two groups. However, for displaced fractures, the LFNS of the FCS is significantly lower than that of the TCS, which may reduce the occurrence of STIT and improve the Harris hip score.

## Introduction

In the past 30 years, the reoperation rate for hip fractures was 10.0–48.8%, and the mortality rate for femoral neck fractures was ~20% (1, 2). It has caused a large number of disability and death in patients worldwide, causing a serious socioeconomic burden (3). At present, most femoral neck fractures are treated with surgery, but the best fixation method remains controversial (4). Biomechanical tests have shown that dynamic hip screw (DHS) can provide reliable stability, especially for displaced and unstable fractures (5), and three cannulated screws (TCS), as a sliding fixation, are more minimally invasive but not sufficiently stable. There have been studies discussing the advantages and disadvantages of the above two fixation methods without clear conclusions (6, 7). We have been trying to use non-sliding fixation (four cannulated screws, FCS) for femoral neck fractures, aiming to improve the stability of internal fixation while maintaining minimally invasive operations.

Currently, there are no comparative clinical studies that discuss the effect of fixation between TCS and FCS. We retrospectively analyzed 102 fresh femoral neck fracture patients who received TCS and FCS treatment in our hospital from January, 2018 to December 2019, and explored the clinical outcomes of the two groups.

This study was approved by the hospital ethics committee (IRB00006761–M2020330), and owing to the retrospective nature of the study, the need for informed consent was waived by the committee.

## Materials and Methods

### Inclusion and Exclusion Criteria

The inclusion criteria were as follows: (1) age ≥18 years; (2) unilateral closed fresh femoral neck fracture (fracture time <3 weeks); (3) use of TCS or FCS for fracture fixation; and (4) follow-up time ≥12 months.

The exclusion criteria were as follows: (1) hip arthritis accompanied by pain and dysfunction symptoms; (2) combined femoral head fracture, femoral shaft fracture, or femoral intertrochanteric fracture; (3) pathological fracture (e.g., primary or metastatic tumor); (4) combined with other diseases or conditions that affect the therapeutic effect, such as metabolic bone disease, sequelae of polio, severe osteoporosis, history of poor bone healing, long-term use of corticosteroids, etc.; (5) >65 years old with Garden III or IV fractures; and 6) history of open reduction.

### Patient Data

We retrospectively analyzed consecutive fresh cases of femoral neck fractures who received TCS and FCS treatment in our hospital from January, 2018 to December, 2019. One hundred and thirteen patients who met the inclusion criteria were finally included, 11 of whom were lost to follow-up. Therefore, 102 patients were followed up for >12 months. Among them, 36 were male and 66 were female, with an average age of 60.9 years (range: 18–86 years). According to the internal fixation method, the patients were divided into the TCS group (60 cases) and the FCS group (42 cases).

### Surgical Methods

Patients received spinal anesthesia or general anesthesia and were positioned supine on the fracture table. Under the guidance of G-arm fluoroscopy, a fracture table was used for closed reduction. The Garden Index (8) was used to assess the quality of fracture reduction. After a successful closed reduction, the routine surgical procedure was followed to implant TCS and FCS (all implants were provided by Depuy Synthes Products, Inc.). For all patients, the first three cannulated screws were implanted in a standard inverted triangle pattern, and the fourth cannulated screw was implanted at an angle not parallel to the other three cannulated screws (**Figure 2**).

### Perioperative Management

All patients received an intravenous injection of the antibiotic cefuroxime (1.5 g) 30 min before the skin incision; another dose was administered approximately 12 h after the operation. Subcutaneous injection of low-molecular heparin for 5 days (40 mg enoxaparin sodium per day) was used to prevent thromboembolism. If patients had been taking anti-osteoporosis drugs before surgery, they were to continue using these drugs. If the patient is newly diagnosed with osteoporosis, anti-osteoporosis drugs should be administered immediately after the operation. Passive and active movements of the hip joint were started on the second postoperative day. It is recommended that patients should perform mainly non-weight-bearing exercises, and exercise with crutches gradually transitioning to partial weight-bearing within 3 months after surgery. Radiographs were reviewed 3 months after the operation. When the fracture line is blurred, patients can start to bear full weight.

### Observation Index

Follow-ups were performed at 1, 3, 6, 12, and 24 months after surgery. The fracture type, blood loss, operation time, hospitalization time, and length of femoral neck shortening (LFNS) were recorded and compared between the two groups. LFNS was measured on an anteroposterior radiograph by comparing the position of the implant (**Figure 2**). This pragmatic method has been used in other studies to measure femoral neck shortening (9). Internal fixation fracture, implant cut-out, and fracture non-union (definition of the U.S. Food & Drug Administration: no obvious sign of fracture healing 9 months after the fracture or 3 consecutive months without significant change in the fracture space) were regarded as internal fixation failure. At the last follow-up, if the patient actively complained that there was soft tissue irritation at the surgical site, it was deemed to have soft tissue irritation of the thigh (STIT). The Harris score was used to assess the hip joint function.

### Statistical Analysis

Statistical analyses were performed using the Statistical Package for the Social Sciences for Windows (version 25.0; IBM Corporation, Chicago, IL, USA). All continuous variables were analyzed using the Shapiro-Wilk test and QQ-plot to determine whether the data were normally distributed. Age and body mass index were normally distributed data, reported as mean ± standard error of mean (x ± s), and the Student's *t*-test was used to evaluate the parameters between the two groups. Other continuous variables were not normally distributed data, expressed as median [interquartile range (IQR)], using the Mann-Whitney U test. Categorical variables are expressed as frequency (percentage) using the Chi-square test and Fisher's exact test. Statistical significance was set at *P* ≤ 0.05.

## Results

A total of 102 patients with an average age of 60.9 (range, 18–86) years were analyzed. The median follow-up time was 25 (22 to 32) months. Except for the Garden classification, there were no significant differences in demographics, injury mechanism, affected side, days from injury to surgery, and follow-up time between the two groups (Table 1).

**Table 1:** Patients characteristics.

	**Total (***n*** = 102)**	**TCS (***n*** = 60)**	**FCS (***n*** = 42)**	***P***
Gender				0.620
Male	36 (35.3)	20 (33.3)	16 (38.1)	
Female	66 (64.7)	40 (66.7)	26 (61.9)	
Age (year)	60.9 ± 17.4	60.9 ± 16.8	60.9 ± 18.4	0.994
Body mass index	22.7 ± 3.4	22.8 ± 2.8	22.6 ± 4.0	0.773
**Involved side**				0.704
Left	56 (54.9)	32 (53.3)	24 (57.1)	
Right	46 (45.1)	28 (46.7)	18 (42.9)	
**Injury mechanism**				0.557
High energy	12 (11.8)	8 (13.3)	4 (9.5)	
Low energy	90 (88.2)	52 (86.7)	38 (90.5)	
DITS (d)	3 (2 to 3)	2.5 (2 to 4)	3 (2 to 3)	0.203
**Garden classification**				0.008
Non-displaced	64 (62.7)	44 (73.3)	20 (47.6)	
Displaced	38 (37.3)	16 (26.7)	22 (52.4)	
**Pauwels classification**				0.125
I	19 (18.6)	15 (25.0)	4 (9.5)	
II	50 (49.0)	26 (43.3)	24 (57.2)	
III	33 (32.4)	19 (31.7)	14 (33.3)	
Follow-up time (month)	25 (22 to 32)	25 (23 to 32)	25 (21.75 to 30.25)	0.269

*Data are presented as mean ± SD, n (%), or median (interquartile range). DITS, days from injury to surgery*.

**Figure 1:**
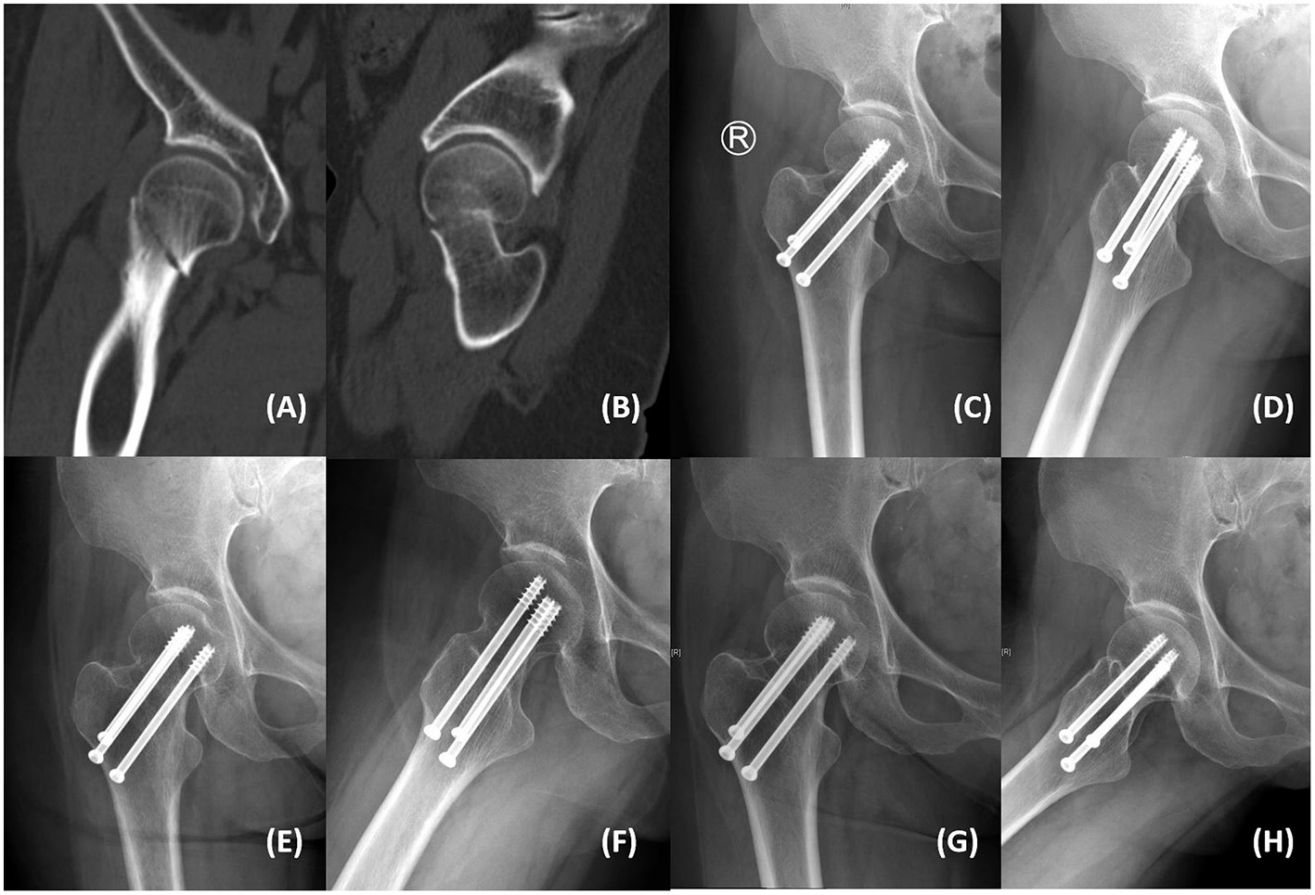
A 59-year-old woman from the TCS group presented with right hip pain and limited mobility after a fall. **(A,B)** Computed tomography showed right femoral neck fracture (Garden III, Pauwels III). **(C,D)** Postoperative anteroposterior and lateral radiographs showed satisfactory fracture reduction with acceptable implant position. **(E,F)** Anteroposterior and lateral radiographs at the 3-month follow-up showed that the fracture line was blurred. **(G,H)** Anteroposterior and lateral radiographs at the 24-month follow-up showed that the fracture was healed without femoral head collapse and internal fixation failure. The Harris hip score was 94 without femoral neck shortening.

**Figure 2:**
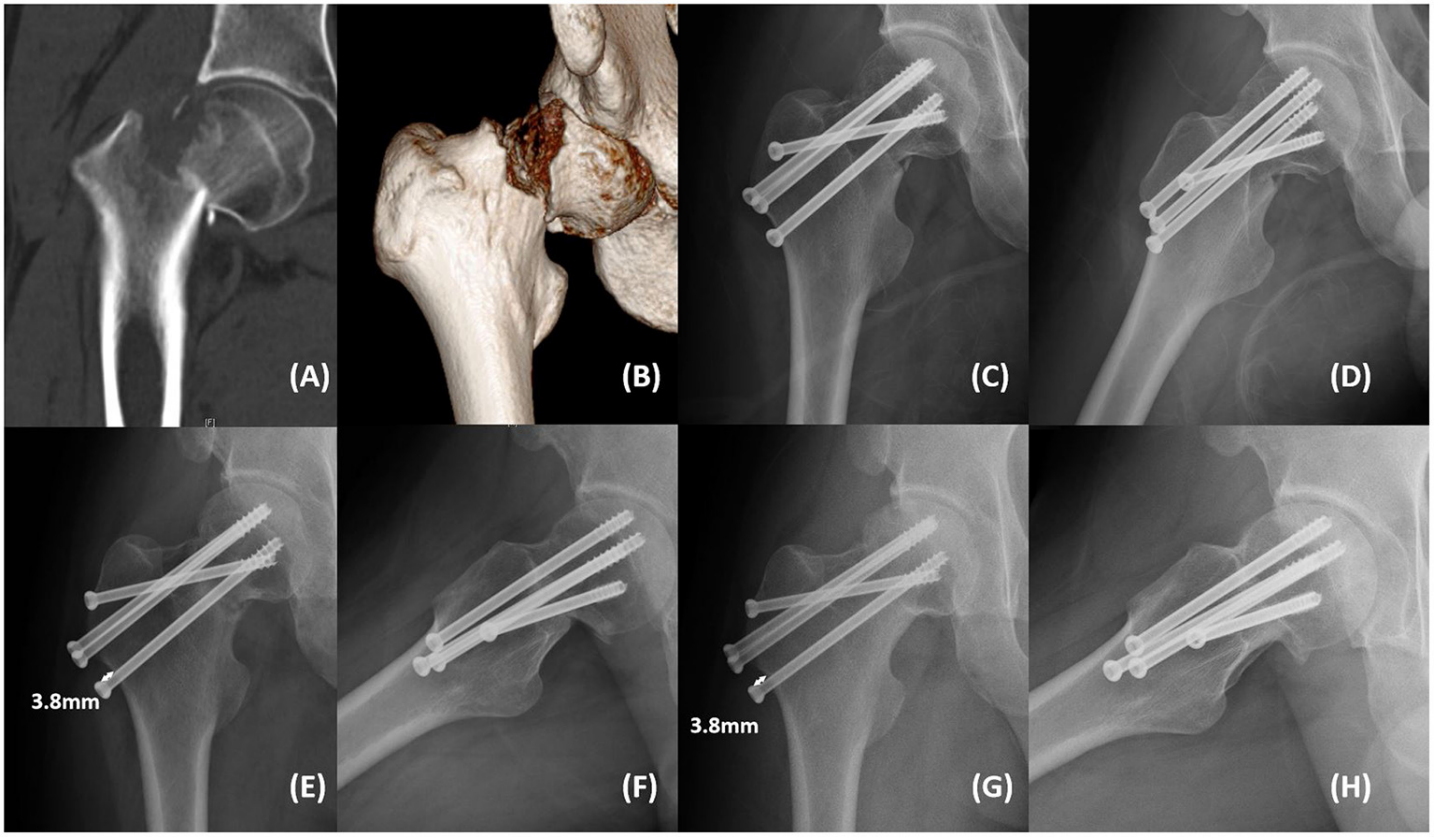
A 28-year-old man from the FCS group presented with right hip pain and limited mobility after a fall. **(A,B)** Computed tomography showed right femoral neck fracture (Garden IV, Pauwels III). **(C,D)** Postoperative anteroposterior and lateral radiographs showed satisfactory fracture reduction with acceptable implant position. **(E,F)** Anteroposterior and lateral radiographs at the 3-month follow-up showed that the fracture line was blurred with femoral neck shortening (3.8 mm). **(G,H)** Anteroposterior and lateral radiographs at the 22-month follow-up showed that the fracture was healed without femoral head collapse and internal fixation failure. The Harris hip score was 97 with mild femoral neck shortening (3.8 mm).

Only one patient in the FCS group experienced dissatisfied reduction (garden index III), and this patient had an internal fixation cut out in the third month after surgery. All other patients achieved satisfactory reduction (Garden Index I), and there was no significant difference in the quality of reduction between the two groups. At the last follow-up, three patients (5.0%, 3/60) in the TCS group experienced internal fixation failure (fracture non-union), and two cases (4.8%, 2/42) in the FCS group experienced internal fixation failure (one cut out and one fracture non-union), and there was no significant difference in the internal fixation failure rate (IFFR) between the two groups (Table 2). There were no significant differences in the hospitalization time, operation time, blood loss, STIT, and Harris hip scores between the two groups, and no avascular necrosis of the femoral head was found. Although there was no significant difference in the number of cases with femoral neck shortening (FS) >5 mm between the two groups, the LFNS of the FCS group was significantly lower than that of the TCS group (*P* < 0.05, Table 2). In the FCS group, only one patient (2.4%, 1/42) had FS >10 mm with claudication and STIT, and the fracture was judged to be non-union at the 9-month follow-up. Similarly, in the TCS group, three patients (5.0%, 3/60) had FS >10 mm, with various degrees of claudication and STIT, and two of them had fracture non-union. In addition, none of the patients had surgical complications such as wound infection, deep infection, deep vein thrombosis, pulmonary embolism, and cardiovascular and cerebrovascular accidents. Two typical cases are shown in Figures 1, 2.

**Table 2:** Clinical and radiological parameters at the latest follow-up.

	**Total (***n*** = 102)**	**TCS (***n*** = 60)**	**FCS (***n*** = 42)**	***P***
Hospitalization time (d)	3 (2 to 4)	3 (2 to 4)	2 (2 to 3.3)	0.310
Operation time (min)	46 (38 to 61)	46 (33 to 61)	43 (38.8 to 60.8)	0.634
Blood loss (mL)	30 (20 to 30)	27.5 (20 to 30)	30 (20 to 30)	0.601
LFNS (mm)	2.4 (1.1 to 5.1)	2.8 (1.8 to 5.2)	1.2 (0.2 to 3.9)	0.001
Harris hip score	92 (85 to 97.5)	92 (84.3 to 99)	92 (86.8 to 96.3)	0.989
IFFR	5 (4.9)	3 (5.0)	2 (4.8)	1.000
FS >5mm	25 (24.5)	16 (26.7)	9 (21.4)	0.545
STIT	25 (24.5)	17 (28.3)	8 (19.0)	0.283

*Data are presented as n (%) or median (interquartile range). LFNS, length of femoral neck shortening; IFFR, internal fixation failure rate; FS, femoral neck shortening; STIT, soft tissue irritation of the thigh*.

Subgroup analysis was performed according to Garden classification (Table 3). For both non-displaced fractures (Garden I/II) and displaced fractures (Garden III/IV), there were no significant differences in hospitalization time, operation time, surgical blood loss, and IFFR between the two groups. For non-displaced fractures, only LFNS in the FCS group was significantly lower than that in the TCS group (*P* < 0.05). However, for displaced fractures, the LFNS, STIT, and Harris hip scores of the FCS group were significantly better than those of the TCS group (*P* < 0.05).

**Table 3:** Comparison of displaced and non-displaced femoral neck fractures at the latest follow-up.

	**Non-displaced**		***P***	**Displaced**		***P***
	**TCS (***n*** = 44)**	**FCS (***n*** = 20)**		**TCS (***n*** = 16)**	**FCS (***n*** = 22)**	
Hospitalization time (d)	3 (2 to 3)	2 (2 to 3)	0.129	4 (3 to 4.8)	3 (2 to 4)	0.151
Operation time (min)	43 (31 to 55)	42 (38 to 56)	0.602	65 (39.8 to 86.8)	50 (39 to 67)	0.138
Blood loss (mL)	22.5 (20 to 30)	27.5 (20 to 30)	0.364	30 (21.3 to 33.8)	30 (20 to 30)	0.540
Harris hip score	96 (85 to 99)	93 (87 to 99)	0.791	89 (74 to 91.8)	91.5 (86 to 96)	0.035
LFNS (mm)	2.5 (1.5 to 3.5)	1.2 (0.2 to 3.5)	0.037	5.7 (2.1 to 9.3)	1.2 (0.3 to 5.0)	0.003
IFFR	1 (2.3)	0	1.000	2 (12.5)	2 (9.1)	1.000
STIT	10 (22.7)	5 (25.0)	0.842	7 (43.8)	3 (13.6)	0.037

*Data are presented as n (%) or median (interquartile range). LFNS, length of femoral neck shortening; IFFR, internal fixation failure rate; STIT, soft tissue irritation of the thigh*.

## Discussion

More than 50% of hip fractures were femoral neck fractures, and which operation method is the best is still controversial (10–12). Internal fixation of fractures and hip replacement are widely used. Liu et al. (13) proposed that the patient's age, bone density, and other information can be quantified, and then the quantified score can be used for surgical decision-making for femoral neck fractures. In general, internal fixation is suitable for patients <65 years of age, and joint replacement is recommended for patients >70 years of age. It can be seen that there is still no clear recommendation for the surgical repair method of femoral neck fracture for patients between 65 and 70 years old (11, 12). In our medical center, only patients >65 years old with Garden III and IV fractures are eligible for hip replacement surgery. Internal fixation is the first choice for young and elderly patients with Garden I and II fractures. Therefore, patients aged >65 years with Garden III and IV fractures were not included in this study.

TCS and DHS are the most common internal fixation implants for femoral neck fractures (14). Compared with DHS, TCS has insufficient biomechanical stability (15–17), but TCS has the advantages of minimally invasive implantation and less blood loss (18). Femoral neck system (FNS) is a slidable internal fixation device with a fixed angle, which is now becoming the standard of care for screw fixation. Compared with DHS, FNS has less damage during the insertion process and also minimizes the required surgical incision length owing to its compact design. Stoffel et al. (15) showed that in an unstable femoral neck fracture model, the biomechanical stability of FNS was twice that of TCS, and its rotational stability was 150% higher than that of TCS, and the biomechanical stability of FNS was comparable to that of DHS. However, TCS and FCS maybe more cost effective. At present, no related literature has compared FNS with FCS and TCS in terms of long-term clinical outcomes and we are trying to summarize our clinical data about FNS. In our study, the operation time, intraoperative blood loss, and hospitalization time in the FCS group were not significantly different from those in the TCS group. FCS aims to increase the stability of internal fixation while retaining the minimally invasive advantages of TCS. Lin et al. (19) reported that in finite element analysis, a modified F-technique configuration (similar to our FCS technique) showed a better performance in resisting shearing and rotational forces in treating Pauwels type III femoral neck fracture compared to those using traditional inverted triangular TCS. However, few studies have compared the clinical results of FCS with TCS. This study compared the clinical data of 102 patients with fresh femoral neck fractures treated with FCS and TCS. We discussed and analyzed the differences in outcomes of the two internal fixation methods in actual clinical applications.

A Study has proposed that the degree of FS can be divided into mild ( ≤ 5 mm), moderate (shortened 5–10 mm) and severe (>10 mm), which may affect the prognosis of hip joint function (9). The heavier the FS degree, the more the implanted cannulated screw protrudes from the bone surface, which may cause STIT in the surgical site and reduce the Harris hip score. Zlowodzki et al. (20) reported that after TCS fixation, the femoral neck was shortened by an average of 10 mm. An international multicenter randomized controlled trial (FAITH trial) (7) reported that approximately 29% of patients had an FS >5 mm after TCS fixation. In our study, patients with FS >5 mm accounted for 21.4 (9/42) and 26.7% (16/60) of the FCS and TCS groups, respectively. At the last follow-up, although there was no significant difference in the proportion of FS >5 mm between the two groups, there were significant differences in LFNS (median 1.2 mm in the FCS group and 2.8 mm in the TCS group). Four patients had FS >10 mm (one and three in the FCS and TCS groups, respectively), all of whom were displaced and Pauwels type III fractures. Although the number of displaced fractures in the FCS group was significantly higher than that in the TCS group, there were no significant differences in the failure rate of internal fixation, Harris hip score, and STIT between the two groups. When performing subgroup analysis according to the Garden classification, it was found that the LFNS, STIT, and Harris hip scores of the FCS group were significantly better than those of the TCS group. It can be seen that FCS has a certain effect on reducing the excessive shortening of the femoral neck, which may reduce the occurrence of STIT and improve Harris hip score.

The non-union rate of femoral neck fractures ranged from 7–33% (21–23). Insufficient blood supply and/or biomechanical instability is closely related to fracture non-union. The FAITH trial (7) reported that the fracture non-union rate after TCS fixation was 6%. Kumar et al. (24) reported that for young patients with displaced femoral neck fractures, the non-union rate after TCS fixation was approximately 13%. In our study, the non-union rate of fracture in the FCS group was 2.4% (1/42) and that in the TCS group was 5.0% (3/60). There was no significant difference in the non-union rates between the two groups.

The incidence of avascular necrosis of the femoral head is approximately 10–30% (21, 23, 25, 26), which may be caused by reduced arterial inflow and venous outflow (27). Kumar et al. (24) reported that the incidence of avascular necrosis of the femoral head after TCS fixation was approximately 7%, while the FAITH test (7) reported an incidence of ~5%. In our study, no avascular necrosis of the femoral head was found at the last follow-up, and the follow-up time still needs to be extended for further observation.

The timing of surgery for the treatment of femoral neck fractures through internal fixation remains controversial. In the past, some researchers believed that femoral neck fractures should be operated on as soon as possible, even within 8 h after injury (28). Upadhyay et al. (29) reported that as long as the femoral neck fracture was internally fixed within 1 week, there was no significant difference in prognosis. Our hospital strives to perform surgical treatment in patients with femoral neck fracture within 48 h after admission, but some patients have not been sent to the hospital in time after injury. In this study, the median time from injury to surgery was 3 days in the FCS group and 2.5 days in the TCS group. There were no significant differences between the two groups. Therefore, the interference of the timing of surgery on the prognostic results of this study can be ruled out.

This study has certain limitations. As a retrospective study, there may have been selection bias, and the sample size was small. Therefore, the results of this study need to be further verified by multi-center, large-sample, prospective randomized controlled trials. Second, this study had a short follow-up time, could only discuss short-term outcomes, and could not evaluate long-term results such as avascular necrosis of the femoral head. Therefore, it is necessary to extend the follow-up time to obtain more accurate conclusions.

## Conclusion

TCS and FCS are effective for femoral neck fractures. For non-displaced fractures, there was no significant difference in the clinical outcomes between the two groups. However, for displaced fractures, the LFNS of the FCS is significantly lower than that of the TCS, which may reduce the occurrence of STIT and improve the Harris hip score.

## Data Availability Statement

The original contributions presented in the study are included in the article/supplementary material, further inquiries can be directed to the corresponding author/s.

## Ethics Statement

The studies involving human participants were reviewed and approved by Peking University Third Hospital Medical Science Research Ethics Committee. Written informed consent for participation was not required for this study in accordance with the national legislation and the institutional requirements.

## Author Contributions

FZ designed the study. XX and YL was responsible for the data collection and analysis and wrote the manuscript. ZC and JF were responsible for the data collection. YT, HJ, ZZ, YG, ZY, and GH were responsible of data analysis and interpretation. All authors have read and approved the final manuscript.

## Funding

This study was supported by National Key R&D Program of China (No. 2018YFF0301102), National Natural Science Foundation of China (No. 81702127), Natural Science Foundation of Beijing (No. 7202222), and Clinical Medicine Foundation of Peking University (No. PKU2020LCX001). All the funding sources were not involved in the design, collection, analysis, and interpretation of the data, or in the writing of the manuscript.

## Conflict of Interest

The authors declare that the research was conducted in the absence of any commercial or financial relationships that could be construed as a potential conflict of interest.

## Publisher's Note

All claims expressed in this article are solely those of the authors and do not necessarily represent those of their affiliated organizations, or those of the publisher, the editors and the reviewers. Any product that may be evaluated in this article, or claim that may be made by its manufacturer, is not guaranteed or endorsed by the publisher.

## References

[1] Bhandari M Devereaux PJ Swiontkowski MF Tornetta P III Obremskey W Koval KJ. Internal fixation compared with arthroplasty for displaced fractures of the femoral neck a meta-analysis. J Bone Joint Surg Am. (2003) 85:1673–81. 10.2106/00004623-200309000-00004 12954824

[2] Mundi S Pindiprolu B Simunovic N Bhandari M. Similar mortality rates in hip fracture patients over the past 31 years. Acta Orthop. (2014) 85:54–9. 10.3109/17453674.2013.878831 24397744 PMC3940992

[3] Zielinski SM Bouwmans CA Heetveld MJ Bhandari M Patka P Van Lieshout EM. The societal costs of femoral neck fracture patients treated with internal fixation. Osteoporos Int. (2014) 25:875–85. 10.1007/s00198-013-2487-2 24072404

[4] Bhandari M Devereaux PJ Tornetta P III Swiontkowski MF Berry DJ Haidukewych G. Operative management of displaced femoral neck fractures in elderly patients An international survey. J Bone Joint Surg Am. (2005) 87:2122–30. 10.2106/JBJS.E.00535 16140828

[5] Jones HW Johnston P Parker M. Are short femoral nails superior to the sliding hip screw? A meta-analysis of 24 studies involving 3,279 fractures. Int Orthop. (2006) 30:69–78. 10.1007/s00264-005-0028-0 16496147 PMC2532072

[6] Hoshino CM O'Toole RV. Fixed angle devices vs. multiple cancellous screws: what does the evidence tell us? Injury. (2015) 46:474–7. 10.1016/j.injury.2014.12.008 25655212

[7] Fixation using Alternative Implants for the Treatment of Hip fractures I. Fracture fixation in the operative management of hip fractures (FAITH): an international, multicentre, randomised controlled trial. Lancet. (2017) 389: 1519–27. 10.1016/S0140-6736(17)30066-1 28262269 PMC5597430

[8] Karanicolas PJ Bhandari M Walter SD Heels-Ansdell D Sanders D Schemitsch E. Interobserver reliability of classification systems to rate the quality of femoral neck fracture reduction. J Orthop Trauma. (2009) 23:408–12. 10.1097/BOT.0b013e31815ea017 19550226

[9] Felton J Slobogean GP Jackson SS Della Rocca GJ Liew S Haverlag R. Femoral neck shortening after hip fracture fixation is associated with inferior hip function: results from the FAITH trial. J Orthop Trauma. (2019) 33:487–96. 10.1097/BOT.0000000000001551 31464855

[10] Luttrell K Beltran M Collinge CA. Preoperative decision making in the treatment of high-angle “vertical” femoral neck fractures in young adult patients. An expert opinion survey of the Orthopaedic Trauma Association's (OTA) membership. J Orthop Trauma. (2014) 28:e221–5. 10.1097/BOT.0000000000000080 25148589

[11] Heetveld MJ Rogmark C Frihagen F Keating J. Internal fixation vs. arthroplasty for displaced femoral neck fractures: what is the evidence? J Orthop Trauma. (2009) 23:395–402. 10.1097/BOT.0b013e318176147d 19550224

[12] Yih-Shiunn L Chien-Rae H Wen-Yun L. Surgical treatment of undisplaced femoral neck fractures in the elderly. Int Orthop. (2007) 31:677–82. 10.1007/s00264-006-0243-3 17033764 PMC2266644

[13] Liu YJ Xu B Li ZY Zhang Q Zhang YZ. Quantitative score system for the surgical decision on adult femoral neck fractures. Orthopedics. (2012) 35:e137–43. 10.3928/01477447-20120123-09 22310396

[14] Bhandari M Swiontkowski M. Management of acute hip fracture. N Engl J Med. (2017) 377:2053–62. 10.1056/NEJMcp1611090 29166235

[15] Stoffel K Zderic I Gras F Sommer C Eberli U Mueller D. Biomechanical evaluation of the femoral neck system in unstable Pauwels III femoral neck fractures: a comparison with the dynamic hip screw and cannulated screws. J Orthop Trauma. (2017) 31:131–7. 10.1097/BOT.0000000000000739 27755333

[16] Li J Zhao Z Yin P Zhang L Tang P. Comparison of three different internal fixation implants in treatment of femoral neck fracture-a finite element analysis. J Orthop Surg Res. (2019) 14:76. 10.1186/s13018-019-1097-x 30871584 PMC6419341

[17] Ye Y Chen K Tian K Li W Mauffrey C Hak DJ. Medial buttress plate augmentation of cannulated screw fixation in vertically unstable femoral neck fractures: surgical technique and preliminary results. Injury. (2017) 48:2189–93. 10.1016/j.injury.2017.08.017 28818323

[18] Ma JX Kuang MJ Xing F Zhao YL Chen HT Zhang LK. Sliding hip screw vs. cannulated cancellous screws for fixation of femoral neck fracture in adults: a systematic review. Int J Surg. (2018) 52:89–97. 10.1016/j.ijsu.2018.01.050 29471156

[19] Lin S Shang J Xing B Wu B Peng R Wang G. Modified F configuration in the treatment of Pauwels type III femoral neck fracture: a finite element analysis. BMC Musculoskelet Disord. (2021) 22:758. 10.1186/s12891-021-04638-2 34488708 PMC8420054

[20] Zlowodzki M Ayeni O Petrisor BA Bhandari M. Femoral neck shortening after fracture fixation with multiple cancellous screws: incidence and effect on function. J Trauma. (2008) 64:163–9. 10.1097/01.ta.0000241143.71274.63 18188116

[21] Gardner S Weaver MJ Jerabek S Rodriguez E Vrahas M Harris M. Predictors of early failure in young patients with displaced femoral neck fractures. J Orthop. (2015) 12:75–80. 10.1016/j.jor.2014.01.001 25972697 PMC4421021

[22] Damany DS Parker MJ Chojnowski A. Complications after intracapsular hip fractures in young adults. A meta-analysis of 18 published studies involving 564 fractures. Injury. (2005) 36:131–41. 10.1016/j.injury.2004.05.023 15589931

[23] Liporace F Gaines R Collinge C Haidukewych GJ. Results of internal fixation of Pauwels type-3 vertical femoral neck fractures. J Bone Joint Surg Am. (2008) 90:1654–9. 10.2106/JBJS.G.01353 18676894

[24] Kumar S Bharti A Rawat A Kumar V Avasthi S. Comparative study of fresh femoral neck fractures managed by multiple cancellous screws with and without fibular graft in young adults. J Clin Orthop Trauma. (2015) 6:6–11. 10.1016/j.jcot.2014.12.008 26549945 PMC4551457

[25] Hoshino CM Christian MW O'Toole RV Manson TT. Fixation of displaced femoral neck fractures in young adults: fixed-angle devices or Pauwel screws? Injury. (2016) 47:1676–84. 10.1016/j.injury.2016.03.014 27269418

[26] Bhandari M Tornetta P. 3rd Hanson B Swiontkowski MF. Optimal internal fixation for femoral neck fractures: multiple screws or sliding hip screws? J Orthop Trauma. (2009) 23:403–7. 10.1097/BOT.0b013e318176191f 19550225

[27] Levack AE Gausden EB Dvorzhinskiy A Lorich DG Helfet DL. Novel treatment options for the surgical management of young femoral neck fractures. J Orthop Trauma. (2019) 33(Suppl 1):S33–7. 10.1097/BOT.0000000000001368 30540670 PMC6294468

[28] Swiontkowski MF Winquist RA Hansen ST Jr. Fractures of the femoral neck in patients between the ages of 12 and 49 years. J Bone Joint Surg Am. (1984) 66:837–46. 10.2106/00004623-198466060-00003 6736085

[29] Upadhyay A Jain P Mishra P Maini L Gautum VK Dhaon BK. Delayed internal fixation of fractures of the neck of the femur in young adults. A prospective, randomized study comparing closed and open reduction. J Bone Joint Surg Br. (2004) 86:1035–40. 10.1302/0301-620x.86b7.15047 15446534

